# Is a vegetarian diet beneficial for bipolar disorder?

**DOI:** 10.1192/j.eurpsy.2025.1055

**Published:** 2025-08-26

**Authors:** S. Gomes-da-Costa, I. Fernandéz, A. Giménez-Palomo, N. Lopez, Y. Rivas, V. Ruiz, M. T. Pons-Cabrera, G. Anmella, E. Vieta, I. Pacchiarotti

**Affiliations:** 1 Mental health care centre, Fundaciò Vidal i Barraquer; 2 Neurology, Hospital del Mar; 3 Psychiatry, Hospital Clinic Barcelona, Barcelona, Spain

## Abstract

**Introduction:**

Lifestyle factors are being increasingly studied in bipolar disorder (BD) due to their possible effects on both course of disease and physical health.

**Objectives:**

The aim of this study was to jointly describe and explore the interrelations between diet patterns, exercise, pharmacological treatment with course of disease and metabolic profile in BD.

**Methods:**

The sample consisted of 66 euthymic or mild depressive individuals with BD. Clinical and metabolic outcomes were assessed, as well as pharmacological treatment or lifestyle habits (diet and exercise). Correlations were explored for different interrelations and a factor analysis of dietary patterns was performed.

**Results:**

Adherence to the Mediterranean diet was low, seen in 37.9% of the patients and was positively associated with perceived quality of life. The amount of exercise was negatively associated with cholesterol levels, with 32.8% of participants rated as low active by International Physical Activity Questionnaire. There was a high prevalence of obesity (40.6%) and metabolic syndrome (29.7%). Users of lithium showed the best metabolic profile. Interestingly, three dietary patterns were identified: “vegetarian,” “omnivore” and “Western.” The key finding was the overall positive impact of the “vegetarian” pattern in BD, which was associated with reduced depression scores, better psychosocial functioning, and perceived quality of life, decreased body mass index, cholesterol, LDL and diastolic blood pressure. Nuts consumption was associated with a better metabolic profile.

**Conclusions:**

A vegetarian diet pattern was associated with both, better clinical and metabolic parameters, in patients with BD. Future studies should prioritize prospective and randomized designs to determine causal relationships, and potentially inform clinical recommendations.

**Disclosure of Interest:**

None Declared

